# Effect of tango versus exercise on chemotherapy-induced neuropathy after breast cancer treatment: a Phase II randomized controlled trial

**DOI:** 10.21203/rs.3.rs-10168428/v1

**Published:** 2026-08-05

**Authors:** Lise Worthen-Chaudhari, Patrick Schnell, Courtney Bland, Kristen Lantis, Jackie Wilder, Karen Hock, Aya Alwan, Ashley Hawke, Gilbert Bader, Ashley P. Davenport, Margaret E. Gatti-Mays, Kai C. Johnson, Dionisia M Quiroga, Sagar D. Sardesai, Daniel G. Stover, Robert Wesolowski, Nicole O. Williams, Jewel E. Crasta, Madeleine E. Hackney, Maryam B. Lustberg

**Affiliations:** 1 Department of Physical Medicine and Rehabilitation, College of Medicine, The Ohio State University, Columbus, OH;; 2 Comprehensive Cancer Center – The James, The Ohio State University, Columbus, OH;; 3 College of Public Health, Division of Biostatistics, The Ohio State University, Columbus, OH;; 4 School of Health and Rehabilitation Sciences, The Ohio State University, Columbus, OH;; 5 Department of Medicine, Emory University, Atlanta, GA;; 6 Center for Visual and Neurocognitive Rehabilitation, Department of Veterans’ Affairs, Atlanta, GA;; 7 School of Medicine, Yale University, New Haven, CT

**Keywords:** chemotherapy-induced neuropathy, rehabilitation, survivorship, dance, music

## Abstract

**BACKGROUND:**

Up to 80% of individuals with breast cancer treated with chemotherapy develop chemotherapy-induced neuropathy (CIN), a dose-limiting neurotoxic response that degrades postural control, sensory function, and quality of life. To evaluate nonpharmacologic solutions for neuromotor dysfunction among individuals with breast cancer and CIN, we performed a randomized controlled trial of two physical activity protocols: Tango Dance (Tango) and Home Exercise (control).

**METHODS:**

We enrolled 51 survivors (mean (SD) = 61.2(9.74) years; female: n=50; male: n=1) with breast cancer, CIN, and postural control deficits. We randomized participants 1:1 into Tango (n=25) or Home Exercise (n=26) intervention arms and conducted both interventions for 8 weeks, 2x per week, for up to 60 minutes per class. Postural control function was measured with eyes closed (QEC) and center of pressure (COP) variables calculated included postural sway amplitude (RMSr (primary), RMSx), sway area (COPa). Additionally, we evaluated patient-reported symptoms, clinical function, participation barriers, and adverse events.

**RESULTS:**

Function, neuropathy symptoms, and fatigue improved with both Tango and Home Exercise intervention. Tango alone evidenced retention of postural control improvements after intervention end (RMSr, p=0.0455; RMSx, p=0.0165; COPa, p=0.0183). Tango alone improved Common Terminology Criteria for Adverse Events patient-reported (PRO-CTCAE) symptoms at 4 (p=0.0402) and 6 (p=0.0110) weeks of training.

**CONCLUSION:**

Both Tango and Home Exercise improve CIN-related deficits. Tango was superior to Home Exercise in two ways: alleviating symptoms at intervention midpoint and supporting retention of neuromotor gains. Further study is required to identify mechanisms driving these effects.

**TRIAL REGISTRATION:**

NCT05114005 (registered 08/15/2021, completed 04/15/2024)

## I. Introduction

Breast cancer (BC) is the second most common cancer experienced by women^1^ following skin cancers yet the consequences of treatment can be debilitating.^2,3^ Up to 80% of BC survivors treated with chemotherapy develop chemotherapy-induced neuropathy (CIN),^4,5^ a dose-limiting neurophysiological effect of treatment that degrades postural control,^6,7^ sensory function^6^, and quality of life^5^ long into survivorship.^8,9^ Treatment options for these physical effects of cancer treatment remain limited, despite the high prevalence and debilitating consequences.^10^ Current standard treatment for CIN is gabapentin, an oral pain management agent, and duloxetine, an oral depression management agent. However, these pharmacologic solutions are critically limited^11^ by side effects and potential interaction with other drugs,^12^ stigma associated with a known anti-depressant,^13,14^ and failure to address motor function directly. The resulting chronic deficits complicate natural aging processes, including worsening age-related functional deficits and increasing fall risk.^2,9,15^ Survivorship medicine seeks to manage and reverse these negative effects of cancer treatment.^2^

Sensorimotor training has emerged as a promising nonpharmacologic treatment to reduce CIN symptoms,^16–21^ ostensibly through stimulating mitochondrial function,^22^ axonal regeneration^23^ and central plasticity^24^ while improving systemic inflammation.^25^ However, challenges around exercising exist for this population.^26–28^ Challenges remain to motivate habitual participation in, and optimize neurorecovery potential available to, physical activity intervention. One untapped avenue to treat CIN is the activity of partnered Adapted Tango (Tango): a Neurological Dance Training (NDT) modality adapted for persons with mobility deficits^29,30^ that delivers sensorimotor training in the context of rhythmic auditory stimulation,^31,32^ creative engagement,^33–35^ and potential for caregiver inclusion.^36^ The activity of dance differs from exercise in several ways^3^ including the neurophenomena invoked (e.g., entrainment,^37^ chronoception,^37^ neural synchrony^38^), the motor-cognitive integration workload,^39^ and intrinsic motivation.^40^ Based on these mechanistic and behavioral differences between exercise and dance activity forms, we sought to evaluate the effect of Tango vs. home exercise on neuromotor control deficits and patient-reported symptoms.

We previously published feasibility results of this trial.^39^ Here, we report effects of both interventions to inform sample size estimations for future trials and systematic reviews of either intervention. We hypothesized that 8 weeks of Tango training (2x per week) would improve neuromotor function among BC survivors with chronic CIN as well as or better than a similar schedule of home exercise for CIN. To evaluate this hypothesis, we compared the effect of Tango versus a CIN-specific home exercise program (home exercise) on postural control (primary), patient-reported outcomes (PROs), and clinical measures of function among individuals with breast cancer, CIN, and balance deficits. This trial represents the first evaluation of Tango as a management option for CIN.

## II. Methods

This single-center, prospective, Phase II randomized controlled clinical trial was performed in line with the principles of the Declaration of Helsinki. Approval to perform the research as described here and, in more detail, in Lantis et al (2023)^41^ and NCT05114005 (registered 08/15/2021, completed 04/15/2024)^42^ was granted by the Institutional Review Board/Ethics Committee of The Ohio State University. The study was conducted at The Ohio State University, Columbus, OH within an oncology outpatient facility. Investigators obtained consent from each participant.

### A. Participants

Fifty-one enrollees (mean(SD) = 61.2(9.74) years; female: n=50; male: n=1) were randomized 1:1 into Tango (n=25) or home exercise (n=26) intervention arms.^41^ Briefly, we included individuals with breast cancer (any stage) ≥ 40 years old; symptomatic for CIN subsequent to taxane exposure that ended at least 3 months prior; with postural sway characteristics outside of the norm^43^ and excluded those with vestibular deficits; poorly controlled diabetes (hbA1C ≥ 8); participation in physical or occupational therapy for neuropathic symptoms during the study, or contraindication to exercise freely (e.g., herniated vertebral disk). Data were collected and/or managed using (Research Electronic Data Capture (REDCap) tools hosted by The Ohio State University.^44,45^

### B. Procedures

Measures are described briefly below and in detail in Lantis et al., (2023).^41^

#### 1. Primary Outcome:

Postural control of the center of pressure (COP) was measured during a challenging 30 second (s) balance task (i.e. bilateral fixed-width stance with eyes closed).^43,46^ Measures of postural control analyzed were clinically useful to predict future falls and/or detect disease state.^43,47^ The primary outcome, postural control amplitude in the resultant (RMSr) plane of motion, was analyzed^48^ using custom software written in Matlab.

#### 2. Secondary Outcome Measures

- *Postural Control:* We calculated sway amplitude in the frontal plane (RMSx), ^48^ 95% ellipse area (COPa),^48^ mean velocity in the frontal plane (COPvx)^48^ and resultant sample entropy using the increment calculation method (SEI).^49^
- *Patient-reported outcomes (PROs):* We collected the following PROs to assess symptom recall and/or ecological momentary assessment (EMA) of symptoms “right now”.
  - *European Organization for Research and Treatment of Cancer’s Quality of Life Questionnaire, Chemotherapy-Induced Peripheral Neuropathy (CIPN-20)* is a validated 20-item patient reported questionnaire instrument for longitudinal evaluation of neuropathy symptoms induced by chemotherapy.^50^
  - *The Brief Pain Inventory (BPI)* is validated for measurement (0 = asymptomatic; 10 = highest severity) of immediate and retrospective (prior 24 hours) pain and pain interference in our target population.^51^ For safety monitoring, we confirmed scores did not change more than 3 points between administrations.
  - *The Brief Fatigue Inventory (BFI)* is validated for measurement (0 = asymptomatic; 10 = highest severity) of immediate and retrospective (prior 24 hours) self-report of fatigue and fatigue interference in our target population.^52^
  - *The Patient-Reported Outcome version of the Common Terminology Criteria for Adverse Events (PRO-CTCAE)* is validated for measurement of cancer-related symptoms. We queried 21 items across theses domains of health: physical (headache, neck pain, pressure in head, nausea or vomiting, fatigue or low energy), cognitive (concentrating, difficulty remembering, confusion, drowsiness, feeling slowed down, “in a fog”, “don’t feel right”); vestibular/ocular (dizziness, blurred vision, balance problems, light sensitivity, noise sensitivity), and emotional (emotional, irritability, sadness, nervous/anxious).^41^ For safety monitoring, we confirmed emotional domain item scores did not change more than 3 points between administrations.
  - *Barriers to participation:* At the beginning of every research encounter, participants were asked “If you missed a session, please indicate the reason why you were unable to attend (e.g., schedule conflict, transportation, forgot).”
  - *Adverse Events and Serious Adverse Events (AEs/SAEs):* At the beginning per research encounter, participants were asked if they had been sick or injured since we last saw them (AEs) and whether the sickness or injury resulted in hospitalization (SAEs). We alerted treating oncologists to SAEs and unexpected assessment results.
- *Clinical examinations*
  - *Timed Up and Go (TUG):* The TUG is a validated clinical test of function^54^ recommended as a screening tool to identify vulnerability^55^ and fall risk^56^ in populations with cancer. Within the test, individuals stand from a chair with arms, walk 3 meters, turn around, and return to sitting as safely yet fast as possible. The test is completed 3 times, with rest allowed between trials. Administrators time each trial, averaging time in seconds to calculate score per timepoint.
  - *6 minute walk test (6mwt):* The 6mwt is validated to assess exercise capacity among individuals with cancer.^57,58,59^ Starting from a standing position, individuals walk as fast and as safely as possible for 6 minutes, resting as needed.^57^ Administrators measure the distance walked after 6 minutes as the 6mwt distance per timepoint.
- *Other measures as reported previously:* Methods and results for the following measures are reported elsewhere: intervention adherence, TUG-Cog times, dance-to-music dose, ratings of perceived physical and mental exertion, brain activity,^39^ motivation, and satisfaction with intervention.^40^

### C. Interventions

Description and rationale for the interventions tested are provided in Lantis, et al. (2023)^41^ and Supplementary Methods. Briefly, both interventions occurred at a frequency of twice per week for 8-weeks and were monitored for exertion in both the physical and mental domains using the Borg Rating of Perceived Exertion (RPE) scale.^39,60^ Instructional staff were certified and experienced to teach each activity. Frequency of interventions was 2x per week for 8 weeks. Intensity was designed in the physical/mental domains, respectively, as light to moderate/light to moderate (Tango); moderate to high/low (home exercise); and monitored using the Borg rating of perceived exertion (RPE) protocol (6 = lowest; 20 = highest)^41^. Activity dose/time was designed to be 20 minutes (Tango) and 45 minutes (home exercise) per session.^39^ Other details about group instruction of in-person Tango (e.g., tempo of music) and individual home exercise are presented elsewhere^39–41^

### D. Evaluation Timeline

Assessments (i.e., screening, repeated baseline, midpoint, post intervention, and 1 month follow up assessments) were performed in an outpatient clinical setting. We evaluated postural control and PROs for both arms during repeated baseline (up to 4x), after 4 (MID) and 8 weeks of intervention (POST), and again at 1 month follow-up (FU). For the Tango arm, we measured postural control before the start of interventions; we could not match this in-person collection for the home exercise group, instead conducting 1 in-person check of postural control mid-intervention.

### E. Statistical Analysis

Each endpoint was analyzed in the intention-to-treat population using linear mixed models with fixed effects for timepoint, randomized assignment, and baseline value of endpoint measure, and subject-level random intercepts to account for within-subject correlation. Estimated marginal means for each arm-by-timepoint combination are reported as baseline-adjusted estimates. Outcomes were log-transformed when appropriate to meet model assumptions. The primary endpoint of the study was change in postural control (i.e., RMSr) between baseline and post 8 weeks of intervention. Secondary endpoints included change in postural control between baseline and 1 month follow up; change in clinical measures between baseline and post intervention as well as between baseline and 1 month follow up; change in PRO survey responses between baseline and post intervention, 1 month follow up, and 2-week intervals (i.e., 2- ,4- ,6-weeks of intervention), with PRO data collected before the beginning of intervention sessions remotely for those in the Home Exercise group and in person for those in the Tango group.

## III. Results

### A. Study flow

**Figure 1:**
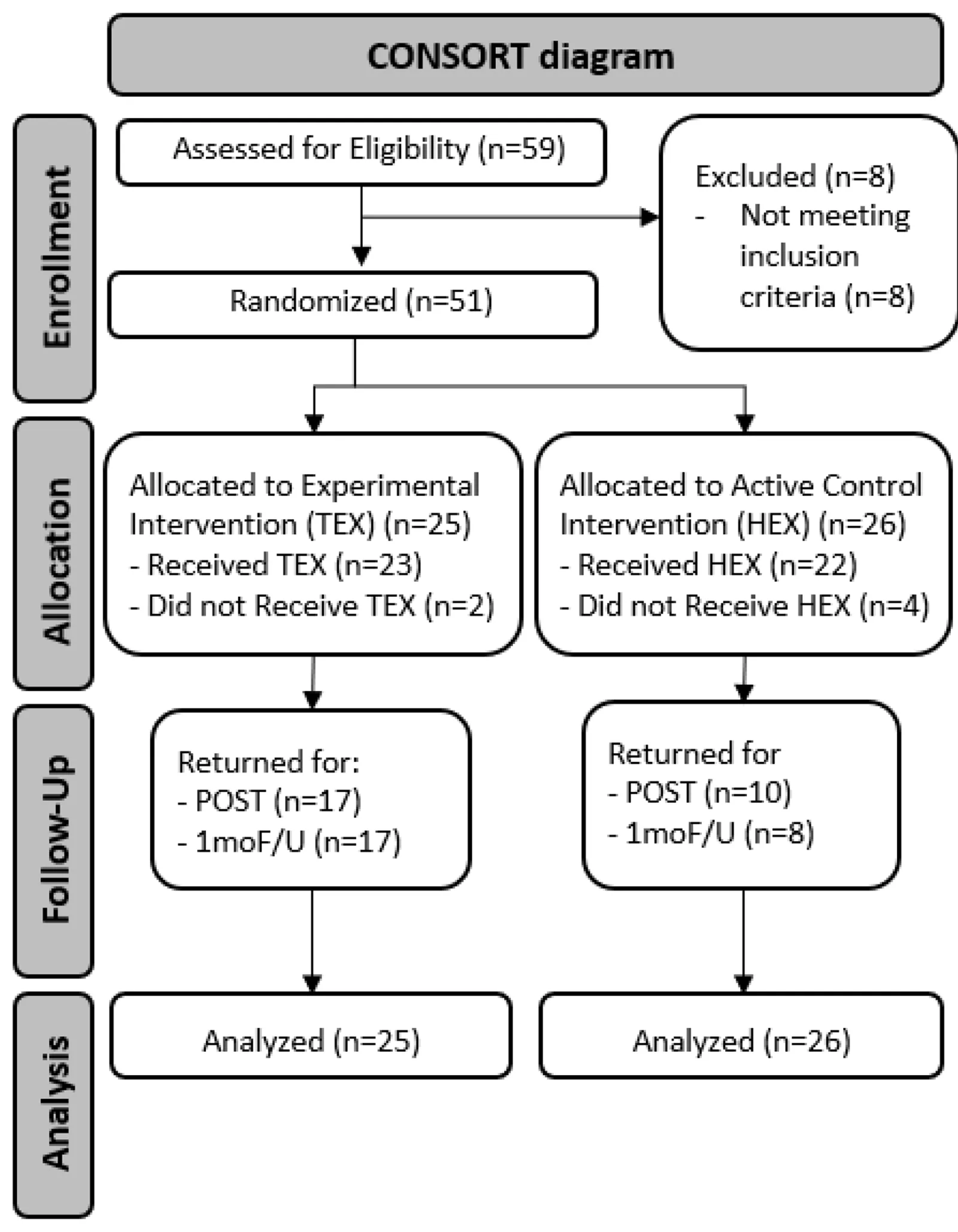
CONSORT diagram.

Intention-to-treat study flow is depicted in Figure 1 per CONSORT recommendations.

### B. Demographics

Participant characteristics were equivalent between randomized groups (Tables I, II).

### C. Intervention Effect

#### 1. Function: Postural control

**Figure 2:**
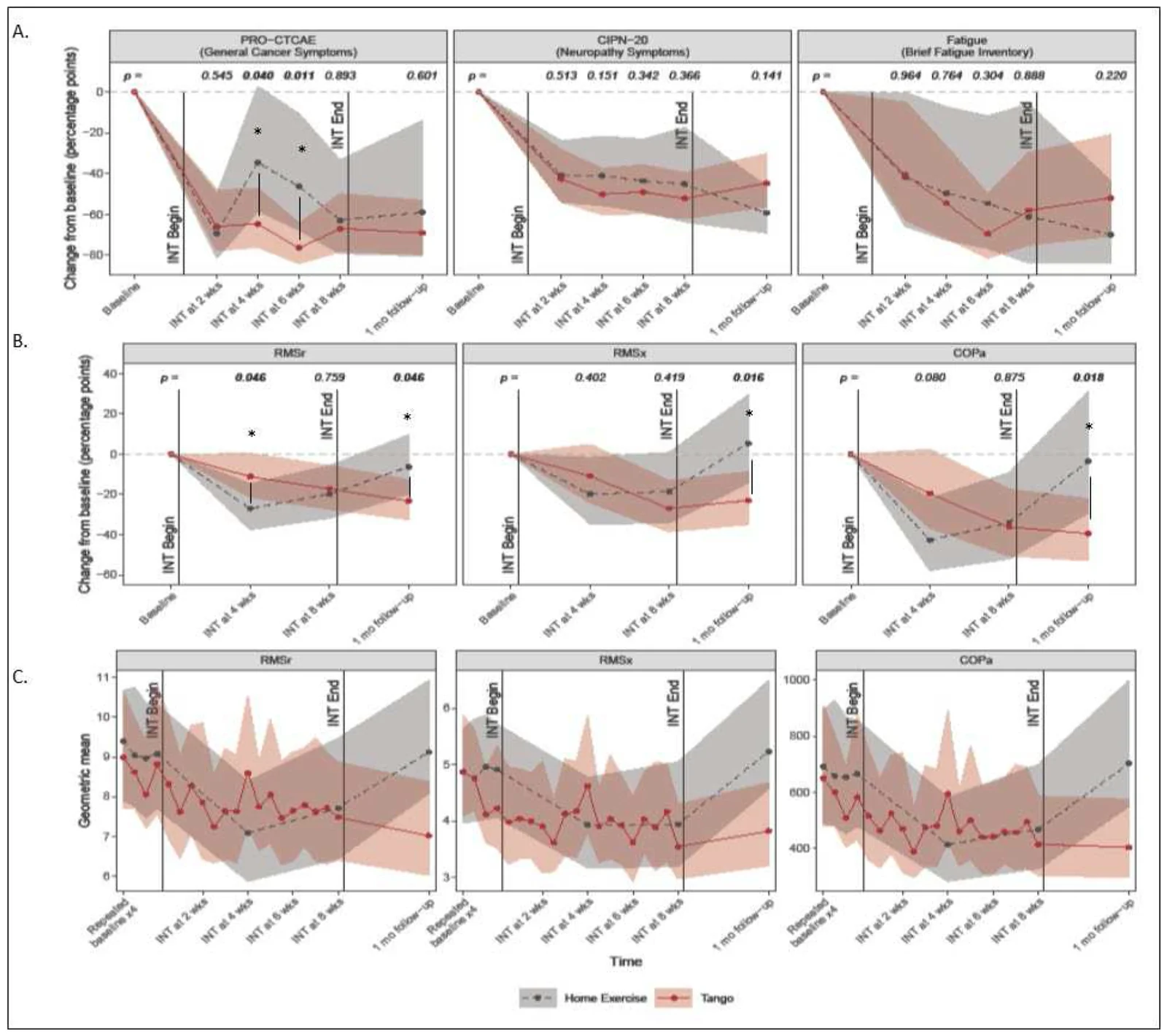
Tango was superior to Home Exercise in (A) general symptoms measured at 4 and 6 weeks of intervention and (B) retention of postural control gains at 1 month follow up post intervention completion. Panel (C) depicts geometric means of raw postural control data collected at the beginning of each tango intervention session to illustrate the dynamics of change associated with tango practice over time. Shaded bands indicate confidence intervals. INT = Intervention. * *p* <0.05.

1. *Primary Outcome Measure:* After 8 weeks of intervention, RMSr improved a comparable amount for individuals randomized to Home Exercise (change in mean log −0.22, 95% CI −0.38 to −0.06, *p* = 0.0076) and Tango groups (change in mean log −0.19, 95% CI −0.32 to −0.06, *p* = 0.0046) (Table 2). At 1 month follow-up, postural control was different between groups (difference in mean log Tango – Home Exercise −0.20, 95% CI −0.40 to −0.004, *p* = 0.0455) with improvements from baseline retained for those randomized to the Tango intervention (change in mean log −0.27, 95% CI −0.39 to −0.14, *p* = 0.0001) and not retained for those randomized to Home Exercise (change in mean log −0.07, 95% CI 0.22 to 0.09, *p* = 0.4) (Table III).
2. *Secondary Measures of Postural Control:* All secondary measures of postural control improved for both groups after 8 weeks of intervention (Table 2). At 1 month follow-up, postural control was different between groups for RMSx (*p* = 0.0165) and COPa (*p* = 0.0183), with improvements retained for those randomized to the Tango group (RMSx *p* = 0.0025; COPa *p* = 0.0001) but not for the Home Exercise group (Figure 2a). Also, at 1 month follow up, neither intervention group retained COPvx training effects but both groups retained SEI training effects (Tango *p* = 0.0002; Home Exercise *p* = 0.0207) (Table III).

### 2. Function: Clinical measures

- *TUG:* As demonstrated in Table III, both groups improved TUG performance after 8 weeks of intervention and retained the improvements at 1 month follow up (*p* <0.0001 all comparisons).
- *6mwt:* As demonstrated in Table III, both groups improved 6mwt performance after 8 weeks of intervention (*p* <0.0001 all comparisons).

#### 3. Symptoms: PROs

- CIN (*CIPN-20):* As demonstrated in Table III, CIPN-20 improved for both groups between baseline and 8 weeks of intervention (Tango *p* < 0.0001; Home Exercise *p* = 0.0041) and was retained for both groups at 1 month follow up (both groups *p* < 0.0001).
- *Pain (BPI):* Neither intervention improved pain severity, however the Tango group demonstrated improvements in pain interference at 8 weeks (*p* = 0.0013) and both groups demonstrated improvement in pain interference at 1month follow up (Tango *p* = 0.0011; Home Exercise *p* = 0.0084; Table III).
- *Fatigue (BFI):* Both groups demonstrated improved fatigue at 8 weeks (Tango *p* = 0.0010; Home Exercise *p* = 0.0363) and at 1 month follow up (Tango *p* = 0.0039; Home Exercise *p* = 0.0002; Table III).
- *Overall symptom burden (PRO-CTCAE)*: Both groups demonstrated improved overall symptom burden at 8 weeks (Tango *p* < 0.0001; Home Exercise *p* = 0.0009) and at 1 month follow up (Tango *p* < 0.0001; Home Exercise *p* = 0.0182; Table III).
- Change per 2 weeks of intervention: Our analysis of change after every 2 weeks of intervention is detailed in Table 4. All variables examined showed improvement within 2 weeks of intervention for both groups. However, Tango retained improvements at 4 and 6 weeks while Home Exercise did not (Figure 2A). We depict postural control response over time in terms of mean(SD) of raw values in Figure 2B. While we do not have comparator data from the Home Exercise arm to analyze between group differences at outside of endpoints reported in Table III, we report data from the beginning of each in-person Tango instructional classes within Figure 2C to provide context regarding the dynamics of neuromotor response over the course of this 8 week activity-based intervention protocol.

#### 4. Barriers to participation

The following barriers to participation in one or more training sessions were reported by participants. We report the barrier followed by the number of enrollees per arm who reported the barrier: time conflict due to work schedule (n=3 Tango, n=3 Home Exercise); travel schedule (n=3 Tango; n=0 Home Exercise); caregiving responsibilities within family (n=4 Tango; n=1 Home Exercise); transportation issues (n=1 Tango; n=0 Home Exercise); forgot (n=1 Tango; n=0 Home Exercise); distance to training location (n=2 Tango only); lost interest (n=2 Tango; n=4 Home Exercise); health (n=4 Tango; n=5 Home Exercise).

#### 5. AEs/SAEs

Four adverse events possibly related to intervention occurred, two within each arm. Two events reported within the Tango group were both rated as mild severity (CTCAE rating = 1). One individual experienced shoulder pain associated with post-surgical dysfunction and one experienced knee pain associated with arthritis. Both events resolved after modification of interventional dance activity by the instructor. For the Exercise group, two events were reported and rated as moderate severity (CTCAE rating = 2). One individual experienced plantar fasciitis and one experienced knee pain associated with arthritis. Despite opportunity to modify home exercise activity per staff instructions, both participants exited the study early due to the moderate AE. No Serious Adverse Events occurred related to either intervention. Events and study team responses to events were deemed acceptable by the NIH-appointed Safety Officer and the IRB of record.

## IV. Discussion

Eight weeks of Tango training improved postural control among BC survivors with chronic CIN as well as a similar schedule of home exercise for CIN. Tango training also produced comparable positive effects to exercise in terms of PROs and clinician tests of function. We conclude that the neurologic dance training modality of partnered Adapted Tango represents a promising physical activity paradigm for the treatment of chronic postural control deficits among individuals with BC and CIN.

Notably, the dose of Tango that improved CIN-related function and sensation was smaller than the doses of alternative physical activity modalities proven effective. The current trial establishes approximately 20 minutes of movement-to-music per class, 2x per week, for 8 weeks as an effective schedule of Tango for CIN^39^, meaning a summed dose of approximately 5 hours total over 2 months. Activity-based treatment options that previously evidenced positive effect for sensory and functional deficits of CIN, in concert, include 10+ hours per month of moderate-vigorous intensity exercise,^61^ low-intensity vibration training,^21^ or yoga^62^. The current finding of relief within less than 3 hours per month provides hope for individuals with cancer that symptom mitigation requires less interventional time than previously recognized.

Tango proved superior to exercise in two ways. Firstly, postural control gains were retained after intervention end. While lifestyle interventions that continue indefinitely are vital options for chronic disease treatment,^63^ ability to stop participating in an intervention for some period of time without loss of prior gains represents an advantage for Tango as a treatment solution. Secondly, participants reported feeling better, sooner, with Tango training in the PRO-CTCAE. While both interventions improved CIN sensation (“since we last saw you”) and fatigue (“in the last 24 hours”) within 4 weeks, only Tango improved PRO-CTCAE items (“right now”) within 4 weeks. Participants randomized to the exercise intervention showed similar gains in general symptom perception once the intervention was over, but not at training midpoint.

Strengths of this study include being the first randomized clinical trial comparing a neurologic dance training modality to exercise among individuals with cancer. The mixed methods combination of patient-reported and functional outcomes is a strength; assessing the effect of intervention in a holistic manner stands the best chance of rapid translation to clinical innovation. Another strength is our clear definition of each interventions in terms of the FITT criteria, which allows for accurate reproduction by others^3^ including ongoing multi-site study of this Tango intervention (R01-AG084676).^64^ Our inclusion of ecological momentary assessment as well as retrospective recall of symptoms is a strength. Momentary and retrospective assessment are capable of detecting different effects among survivors,^65^ therefore, it is relevant to assess symptoms of cancer survivorship using both query paradigms. Future research should consider performing ecological momentary assessment of survivorship symptoms in addition to retrospective recall of symptoms, in the way that validated PROs such as the Brief Pain Inventory and Brief Fatigue Inventory^41^ do.

Limitations of this study include a small sample size. The sample size estimation supporting recruitment decisions was designed to provide sufficient statistical power to assess change in biomechanical outcomes but not in PROs. Additional multi-site study (R01-AG084676) is underway to assess participant perception of effects on symptoms, definitively. Another limitation of this study is that participants who identified as Latina, Asian, and Black were underrepresented relative to those who identified as White. Ongoing studies have addressed this issue by adding intervention delivery sites that increase access for nonwhite populations and by offering Tango instruction in Spanish as well as English.^64^ A final limitation of this study is our inability to differentiate the contributions of social engagement, musical engagement, and sensorimotor skill training. Future research should consider isolating the mechanistic contributions of these different aspects of the successful Tango intervention.

## V. Conclusion

When tailored for individuals with chronic CIN due to BC treatment, the Neurologic Dance Training technique of Tango represents a promising physical activity paradigm for restoring lost function while alleviating sensory symptoms. Additional study is indicated to confirm and optimize these treatment effects.

## Supplementary Material

This is a list of supplementary files associated with this preprint. Click to download.

- R21AG068831Aim1ResultsTableIportrait.docx
- R21AG068831Aim1ResultsTableIIportrait.docx
- R21AG068831Aim1ResultsTableIIIlandscape.docx
- R21AG068831Aim1ResultsSuppAnalysis.docx

Tables I to III are available in the Supplementary Files section.

## Data Availability

The data and source code supporting the analysis and findings of this study are available within the article and/or its supplementary material.

## Take home messages:

- Eight weeks of partnered Tango improved functional deficits and symptoms of CIN as much or more than home exercise.
- The dose of Tango activity that improved CIN deficits across function and symptoms (< 3 hours per month) is the smallest effective dose evidenced to date of activity-based intervention for CIN.
- After 4 weeks of Tango intervention, patient-reported symptoms of physical, cognitive, and emotional health (PRO-CTCAE) improved more than with home exercise.
- One month following intervention conclusion, Tango but not home exercise was associated with retained neuromotor control gains.

## Acronyms:

6mwt: six-minute walk test
BC: Breast Cancer
BFI: Brief Fatigue Inventory
BPI: Brief Pain Inventory
CIN: chemotherapy-induced neuropathy
CIPN-20: European Organization for Research and Treatment of Cancer’s Quality of Life Questionnaire, Chemotherapy-Induced Peripheral Neuropathy survey measure
COP: center of pressure
COPa: center of pressure 95% ellipse area
COPvx: mean medial-lateral velocity of the center of pressure
EMA: Ecological Momentary Assessment
HEX: Home exercise group
NDT: Neurologic Dance Training
OH: Ohio
PRO: patient-reported outcome measure
PRO-CTCAE: Patient-Reported Outcome version of the Common Terminology Criteria for Adverse Events
QEC: quiet standing with eyes closed
RPE: Rating of Perceived Exertion
REDCap: Research Electronic Database Capture platform
RMSr: root-mean square of the resultant center of pressure
RMSx: root-mean square of the medial-lateral center of pressure
SD: standard deviation
SEI: sample entropy calculated using the increment method
TEX: Tango group
TUG: Timed-Up-and-Go test of function
TUGCog: Timed-Up-and-Go test administered with concurrent cognitive task
